# Layered transition metal dichalcogenides: promising near-lattice-matched substrates for GaN growth

**DOI:** 10.1038/srep23708

**Published:** 2016-03-30

**Authors:** Priti Gupta, A. A. Rahman, Shruti Subramanian, Shalini Gupta, Arumugam Thamizhavel, Tatyana Orlova, Sergei Rouvimov, Suresh Vishwanath, Vladimir Protasenko, Masihhur R. Laskar, Huili Grace Xing, Debdeep Jena, Arnab Bhattacharya

**Affiliations:** 1 Department of Condensed Matter Physics and Materials Science, Tata Institute of Fundamental Research, Mumbai, India; 2 UM-DAE Center for Excellence in Basic Sciences, Mumbai, India; 3 Department of Electrical Engineering, University of Notre Dame, Notre Dame, USA; 4 Department of Chemical and Biological Engineering, University of Wisconsin-Madison, USA

## Abstract

Most III-nitride semiconductors are grown on non-lattice-matched substrates like sapphire or silicon due to the extreme difficulty of obtaining a native GaN substrate. We show that several layered transition-metal dichalcogenides are closely lattice-matched to GaN and report the growth of GaN on a range of such layered materials. We report detailed studies of the growth of GaN on mechanically-exfoliated flakes WS_2_ and MoS_2_ by metalorganic vapour phase epitaxy. Structural and optical characterization show that strain-free, single-crystal islands of GaN are obtained on the underlying chalcogenide flakes. We obtain strong near-band-edge emission from these layers, and analyse their temperature-dependent photoluminescence properties. We also report a proof-of-concept demonstration of large-area growth of GaN on CVD MoS_2_. Our results show that the transition-metal dichalcogenides can serve as novel near-lattice-matched substrates for nitride growth.

The group-III nitride semiconductors^1^ — GaN, AlN, InN and their alloys — are important materials for compact, energy-efficient, solid-state lighting sources as well as ideal candidates for high-power^2^/high-temperature^3^ electronic devices. There have been phenomenal improvements in the performance of GaN-based LEDs^4^,^5^, laser diodes^6^, and transistors^7^; however, a fundamental challenge in these materials has been the extreme difficulty of obtaining a native GaN substrate compelling the use of non-lattice-matched substrates like sapphire or silicon^8^,^9^,^10^. While breakthroughs^11^,^12^ in epitaxial growth techniques have dramatically improved material quality on sapphire, there has always been a quest to seek lattice-matched substrate materials for GaN growth. In this work, we show that several layered transition metal dichalcogenides^13^ are closely lattice matched to GaN and report the metalorganic vapour phase epitaxial (MOVPE) growth of GaN on a range of such layered materials. In particular, we show WS_2_ and MoS_2_ to be promising substrates for III-nitride growth.

The past few years have seen a frenzy of activity in research in 2-dimensional layered materials, motivated by the exceptional properties of graphene^14^ and the demonstration of a range of novel devices. The transition-metal dichalcogenides (TMDCs)^13^ — layered materials analogous to graphene but which also offer a bandgap^15^ – have been a subject of great recent interest due to their own unique electrical and optical properties. Most of the work in semiconducting layered materials has focused on their potential for opto-electronic devices^16^,^17^,^18^,^19^,^20^. What has perhaps not been noticed is their relatively close in-plane lattice match with materials of the III-nitride family. Figure 1 shows the bandgap versus in-plane lattice parameter for different III-nitrides and TMDCs. WS_2_ and MoS_2_ have a lattice mismatch of only 1.0% and 0.8%, respectively to the ‘*a*’ lattice parameter of GaN. Further, the weak out-of-plane (van der Waal’s) interactions and absence of dangling bonds on the surface of the layered chalcogenides may help in controlling stress between the nitride layer and substrate. The TMDCs can thus not only provide novel near-lattice-matched substrates for nitride growth, but also likely to allow the study of novel heterostructures in combination with the III-nitride materials.

## Results and Discussion

To test their viability as substrates for nitride growth, mechanically-exfoliated flakes of the layered TMDCs were transferred to SiO_2_ (300 nm thick) coated silicon wafers (details in supplementary information section S2). We chose SiO_2_-coated Si rather than sapphire, Si or SiC to minimize parasitic growth outside the flakes which may hinder the characterization of the GaN layer. In addition, for a proof-of-concept demonstration of large-area growth, CVD MoS_2_ films synthesized by the sulphurization of thin Mo layers were used (details in supplementary information sections S2 and S3).

The heat-up procedure, carrier gas, nucleation conditions, growth temperature and V/III ratio were varied to determine the optimal conditions for growth of GaN on WS_2_ and MoS_2_. The MOVPE growth of GaN typically occurs at a high temperature, >1000 °C, in an ambient of ammonia and hydrogen, rather aggressive conditions, which are in some cases close to the thermal decomposition temperatures of the chalcogenide materials. This makes the procedure for initiation of growth on the TMDC material very critical, especially the choice of carrier gas and temperature ramp during the heat-up procedure, and the conditions for the growth of the nucleation layer. Our observations suggest that ramping up to the growth temperature is best done using nitrogen (N_2_) as a carrier gas to preserve the integrity of the TMDC layer (a detailed study of thermal annealing of MoS_2_ and WS_2_ in different ambients is presented in supplementary information section S4). We switch the carrier gas to hydrogen just before the growth of the GaN layer, and again cool down to room temperature under N_2_. Based on optimization experiments, we have used a short initial GaN nucleation layer grown at 900 °C for 40 s at a V/III ratio of ~1400, following which a second layer is grown at the more usual growth temperature of 1040 °C at a V/III ratio of ~2100 (we will refer this second layer growth time as t_*GaN*_). The low temperature GaN layer also ensures the conformal coverage of GaN on the TMDC substrate.

### Growth of GaN on WS_2_

**Figure 2:**
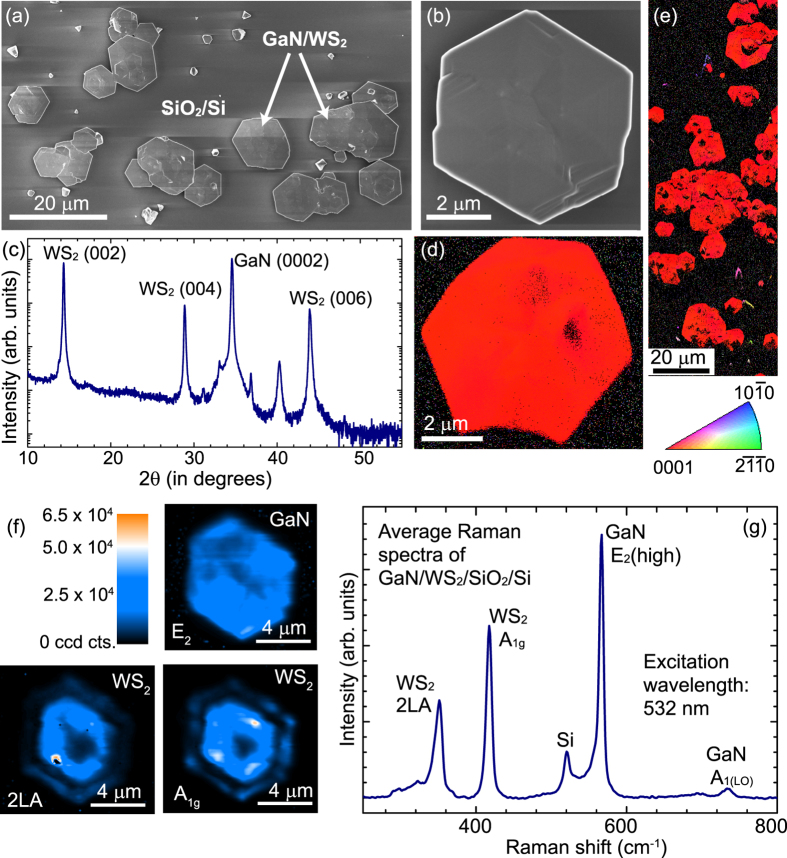
Growth of GaN on exfoliated WS_2_ flakes. (**a**) Scanning electron microscopy confirms near-hexagonal crystals of GaN growing only in the region covered by the WS_2_ flakes. (**b**) Micrograph showing single hexagonal crystal of GaN grown on WS_2_ (**c**) X-ray diffraction profile of GaN on WS_2_ shows preferential (0002) orientation. (**d**,**e**) EBSD maps of GaN grown on exfoliated WS_2_ clearly show that the grown GaN layer is single crystal and (0002) oriented. (**f**) Integrated Raman mapping over an area of 10 *μ*m × 11 *μ*m for the intensity of following Raman modes E_2_(high) of GaN, 2LA and A_1*g*_ mode of WS_2_. The colour scale is based on the intensity of the GaN E_2_(high) mode. Fig. S2 in the supplementary information shows the WS_2_ feature in greater detail confirming its presence everywhere below the GaN layer. (**g**) Spatially averaged Raman scattering spectrum of GaN/WS_2_ over the flake shown in (**f**) shows the survival of WS_2_ after growth and the peak position of E_2_(high) indicates that GaN layer on WS_2_ is strain-free.

We first discuss the growth of GaN on WS_2_. For t_*GaN*_ = 300 s, nearly hexagonal crystals of GaN are obtained only on WS_2_ flakes as shown in Fig. 2(a,b). From the XRD profile of GaN on WS_2_ (Fig. 2(c)), it is clearly seen that GaN is growing in the (0002) direction as expected. The few stray peaks are possibly due to either random nucleation on SiO_2_/Si or due to growth on mis-oriented WS_2_ flakes stamped on the substrate. All the GaN/WS_2_ flakes are oriented in (0002) direction as confirmed from the large area electron back scattering diffraction (EBSD) map (Fig. 2(e)). A detailed EBSD map of one flake (Fig. 2(d)), shows that the GaN is single crystal with no grain boundary. Similar EBSD maps on several other flakes confirm the single crystal growth of GaN on WS_2_. The observation of a (0002) oriented GaN layer is not surprising. Even if there are no bonds across the interface, the atomic arrangement of the underlying layer would still influence the energy landscape on the growth surface and hence have an effect on the nucleation and growth of the epilayer. Thus, the lattice structure of the substrate will influence the epitaxial relation. For closely-lattice-matched systems, as in the case of the TMDCs, the substrate’s lattice points will be the energetically favoured regions and the little strain that may arise from a small mismatch is easily relaxed because of the absence of strong interlayer bonding. The lattice parameters of GaN were calculated from the (0002) and (10
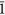
1) peak positions in the powder XRD scan. The obtained value of the in-plane lattice parameter was 3.19 Å which is almost equal to that reported for unstrained GaN i.e. 3.189 Å. Thus, as expected, the GaN layer being grown on a closely-lattice matched substrate is almost strain-free.

Raman spectroscopy was used to examine the residual stress and to monitor the quality of the GaN layer. The frequency shift of the strongest phonon mode E_2_(high) is very sensitive to the residual stress^21^,^22^ whereas the width depends on the quality of the layer^22^. The lattice- and thermal-mismatch between the layer and the substrate result in a residual strain which is usually compressive for GaN grown on sapphire, and tensile for GaN on Si^23^. The E_2_(high) mode of strain-free GaN is known to be at 566.2 cm^−1^ at room temperature^21^, and shifts to higher frequency with compressive strain whereas a downshift indicates tensile strain. Figure 2(f) shows the Raman integrated maps of the E_2_(high) mode of GaN, and 2LA and A_1*g*_ modes of WS_2_. These maps clearly show the hexagonal-shaped islands of GaN grown on the underlying WS_2_ with their edges being parallel to each other. The spatial extent of the GaN may be larger than the underlying WS_2_ flake due to lateral overgrowth at the edges. The homogeneous intensity distribution of the E_2_(high) peak also points to the uniform GaN growth over the WS_2_ flake. The spatially-averaged Raman spectrum of GaN/WS_2_ over a 10 *μ*m × 11 *μ*m area is shown in Fig. 2(g). The E_2_ phonon peak for GaN/WS_2_ is observed at 566.4 cm^−1^ which is almost near the value reported for the unstrained GaN. This also confirms that the GaN layer grown on WS_2_ is nearly strain-free. The linewidth of the E_2_(high) peak is 6 cm^−1^, comparable with that of (0002)-oriented GaN of similar thickness grown on sapphire with the standard two-temperature recipe.

### Growth of GaN on MoS_2_

**Figure 3:**
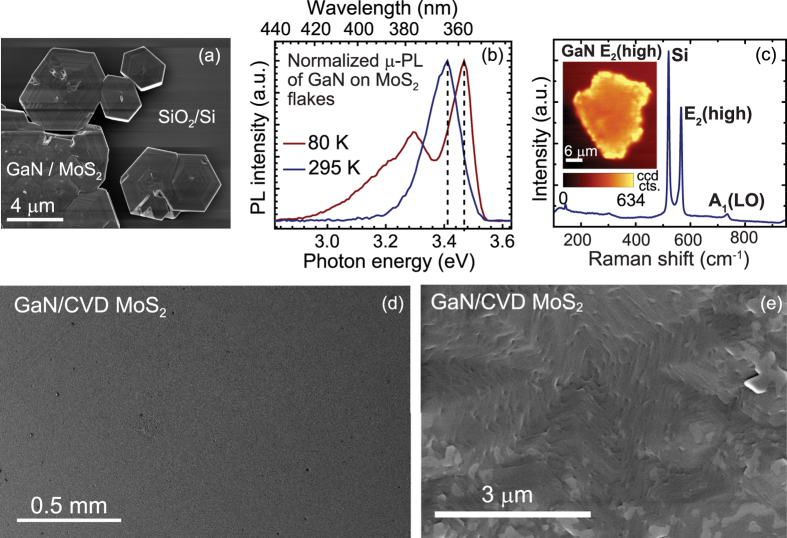
Growth of GaN on MoS_2_. (**a**) Hexagonal crystals of GaN which are obtained only on MoS_2_ flakes (**b**) Room- and low- temperature *μ* - photoluminescence spectra showing strong near-band-edge emission from the GaN (**c**) Spatially averaged Raman spectrum over GaN/MoS_2_ flake (inset shows the integrated Raman map for the intensity of E_2_(high) phonon mode of GaN). (**d**,**e**) SEM images showing the extension of GaN growth to large area CVD MoS_2_.

Following a similar recipe, GaN layers were grown on MoS_2_ as well. While one group had reported the molecular beam epitaxial growth of GaN on bulk MoS_2_ in the 1990s^24^,^25^, there has been no systematic study. GaN layers were deposited on exfoliated MoS_2_ using MOVPE and again nearly hexagonal-shaped crystals of GaN are obtained only on the MoS_2_ flakes. A typical SEM image of the surface is shown in Fig. 3(a). The GaN layer shows strong near-band-edge luminescence in room- and low-temperature photoluminescence (PL) (Fig. 3(b)). The details of PL are discussed later. Once again, the peak shift of the E_2_(high) Raman mode is very small indicating nearly strain-free GaN on MoS_2_ (Fig. 3(c)). The EBSD map of GaN grown on exfoliated MoS_2_ (supplementary information section S5) also shows that grown GaN layer is single crystal similar to GaN grown on WS_2_ and oriented in (0002) direction. From the low magnification and a zoomed-in SEM image of GaN grown on large area CVD MoS_2_ (Fig. 3(d,e)), it is clear that there is conformal coverage across the substrate and this method is scalable to larger substrate sizes.

**Figure 4:**
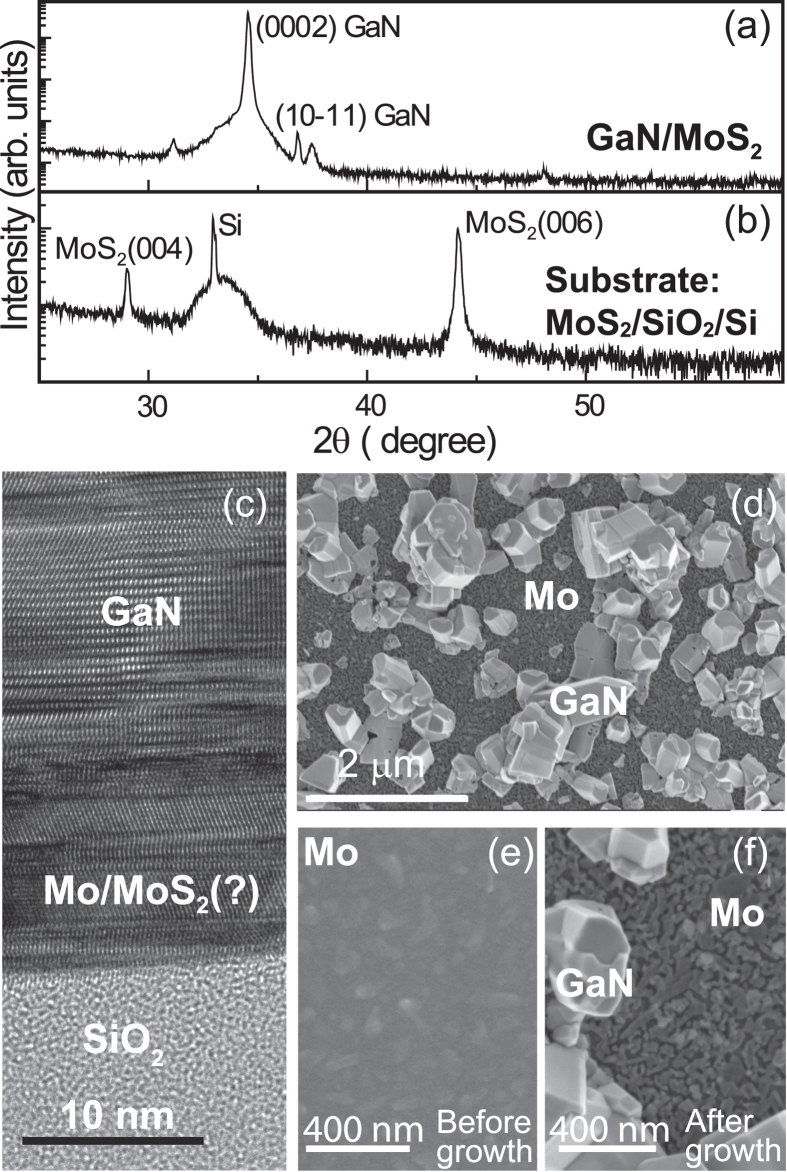
Necessity of MoS_2_ for GaN growth. (**a**) X-ray diffraction profile of GaN on MoS_2_ shows preferential (0002) orientation but no MoS_2_ peak after growth as compared to substrate XRD profile(**b**). (**c**) Cross-sectional transmission electron micrograph does not show MoS_2_ at the interface of substrate and GaN layer. (**d**) The SEM shows faceted chunks of GaN on Mo substrate and no conformal coverage of GaN. The micrographs below (**d**) show sputtered Mo on sapphire before (**e**) and after (**f**) growth.

The GaN layers grown on exfoliated MoS_2_ are preferentially oriented in (0002) direction as seen from XRD pattern in Fig. 4(a) (the XRD profile of GaN growth on CVD MoS_2_ is very similar to that grown on exfoliated MoS_2_, and is shown in supplementary information section S6). However, the surprising observation is that there are no characteristic MoS_2_ peaks after the MOVPE growth (Fig. 4(a,b)). This suggests that the MoS_2_ may have degraded under the MOVPE growth conditions (high growth temperature and exposure to ammonia and hydrogen). TEM imaging was used to confirm this hypothesis. As seen in Fig. 4(c), clear lattice fringes of the GaN layer are present in the top of the image. However, below the GaN, instead of the characteristic signature of layered MoS_2_, only metallic molybdenum (Mo) was detected. A simple experiment to check the role of MoS_2_ in the growth of GaN was to attempt to grow GaN on Mo metal alone using sputtered Mo layers deposited on sapphire. As seen in Fig. 4(d), faceted chunks of GaN were obtained and there was no conformal growth of GaN on Mo. The morphology of the the Mo surface also changed after the MOVPE growth of GaN on Mo (Fig. 4(e,f)). This suggests that the MoS_2_ was indeed present and served as a substrate for the initial growth of the GaN layer, but degraded at some point during the high temperature growth.

### Comparative photoluminescence spectroscopy of GaN grown on WS_2_ and MoS_2_

**Figure 5:**
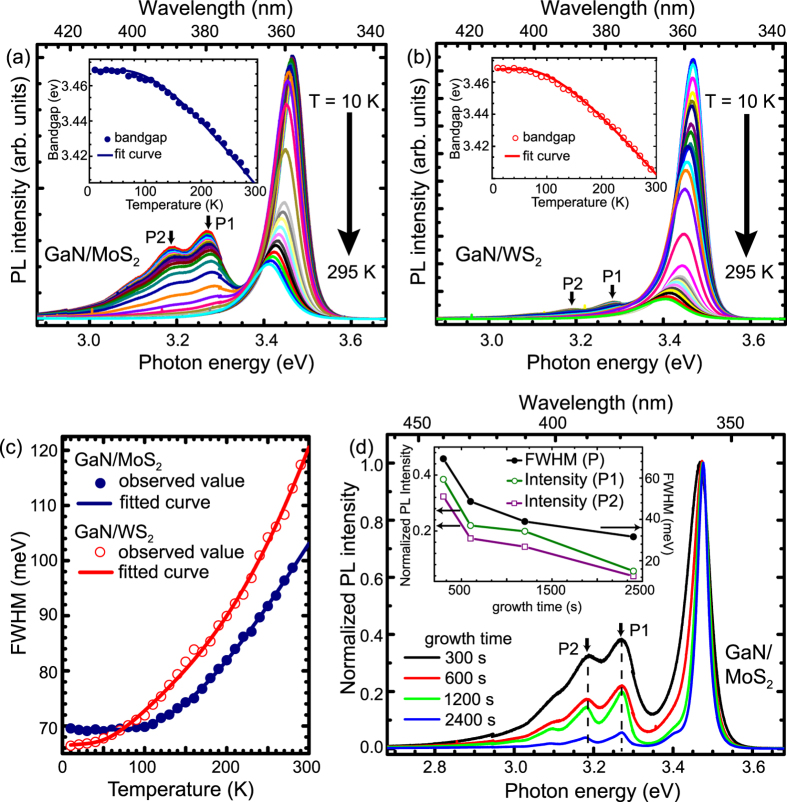
Comparative PL spectroscopy of GaN grown on WS_2_ and on MoS_2_. Temperature dependent photoluminescence of GaN grown (**a**) on WS_2_ and (**b**) on MoS_2_. Insets of (**a**,**b**) show the Bose-Einstein expression fit for the variation of NBE peak positions with temperature. (**c**) Temperature dependence of FWHM of NBE emission line for GaN/WS_2_ and GaN/MoS_2_. (**d**) Low temperature (10 K) PL of GaN grown on MoS_2_ with different t_*GaN*_ (inset shows that intensity of peaks P1 and P2 decreases and NBE emission linewidth decreases with increasing t_*GaN*_).

Temperature-dependent photoluminescence measurements were done on both the GaN/WS_2_ and GaN/MoS_2_ samples as shown in Fig. 5(a,b). The PL spectra exhibit a dominant near-band-edge (NBE) transition band at 3.41 eV (temperature (T) = 290 K), which clearly blue-shifts with decreasing temperature. Apart from this, there are two additional peaks at lower energies at 3.27 eV and 3.18 eV which we label as P1 and P2, respectively. We first discuss the behaviour of the NBE emission. The insets of Fig. 5(a,b) show the variation of NBE peak position with temperature which are fitted by the Bose-Einstein expression^26^:


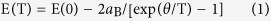


where E(0) is the transition energy at T = 0 K and a_*B*_ is the strength of the average exciton-phonon interaction and *θ* is the average phonon frequency. The values of E(T), a_*B*_ and *θ* as obtained from the fitted curves (with 95% confidence bounds) are shown in Table 1 and are in close agreement with the reported values for this temperature range^27^. The E(0) values obtained for the GaN grown on WS_2_ and MoS_2_ are 3.468 ± 0.002 eV and 3.469 ± 0.002 eV, respectively, very close to 3.471 eV^28^ for unstrained GaN, again indicating strain-free layers.

The FWHM of the NBE emission line increases with increasing temperature due to increasing exciton-phonon interaction at higher temperatures. The temperature dependence of the linewidth has the usual form^29^:


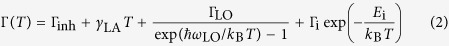


where Γ_inh_ is the inhomogeneous broadening term, *γ*_LA_ is a coefficient of exciton-acoustic-phonon interaction, Γ_LO_ is the exciton-LO-phonon coupling constant, *ω*_LO_ is the LO-phonon energy, Γ_i_ is a proportionality factor which accounts for the concentration of impurity centers and *E*_i_ is the binding energy of impurity-bound excitons averaged over all possible locations of the impurities. The obtained value of Γ_i_ from the fit curves for WS_2_ and MoS_2_ (Fig. 5(c)) are 55 ± 16 meV and 171 ± 87 meV, respectively which indicates that the concentration of impurity centers in GaN grown on WS_2_ is lesser than that for the GaN grown on MoS_2_. The same is also evident from the value of *E*_i_ which is lesser in case of GaN/WS_2_ compared to that for GaN/MoS_2_ (Table 1).

The peak intensity of P1 and P2 is larger in GaN/MoS_2_ layer compared to GaN/WS_2_ layer, which strongly suggests that these peaks arise from the defects originating from the degradation of the substrate. To confirm this hypothesis, GaN layers of different thicknesses were grown on MoS_2_ (t_*GaN*_ = 300 s, 600 s, 1200 s and 2400 s). The low temperature (T = 10 K) PL spectra for these samples, normalized to the NBE peak, are shown in Fig. 5(d). It can be seen that on increasing t_*GaN*_, i.e. with larger thickness of the GaN layer, the intensity of P1 and P2 decreases (inset of Fig. 5(d)). At our laser excitation energy the absorption length in GaN is only ~50–60 nm, hence the PL observed is mainly from the top ~200 nm GaN layer. The decrease in intensity of P1 and P2 thus indicates that the defect density in the top GaN layer reduces as the thickness increases. While this could be attributed to an overall reduction in extended defects, we believe that in our samples, P1 and P2 are related to point defects that originate from the GaN/substrate interface due to MoS_2_ degradation. This was confirmed from the secondary ion mass spectrometry (SIMS) profile which showed a significant concentration of sulphur in the GaN layer (supplementary information section S7). Also, the FWHM of GaN/MoS_2_ decreases on increasing t_*GaN*_ (the inset of Fig. 5(d)), indicating better GaN quality on increasing thickness. However, we have not found luminescence of sulphur impurities in GaN reported in literature.

### GaN grown on other TMDCs

GaN growth on other mechanically-exfoliated TMDCs like WSe_2_, MoSe_2_, ReS_2_ and ReSe_2_ (lattice mismatch to GaN − ~1–3%) was also attempted (SEM images in supplementary information section S8). The micrographs clearly indicate that growth of GaN layer is possible on these substrates. However the heat-up and the growth initiation steps would need to be optimized for the different TMDCs keeping in mind their thermal stabilities and reactivities. With the proper optimization of MOVPE growth conditions, these TMDCs can also be potential substrates for III-nitride growth.

## Conclusion

In conclusion, we report the MOVPE growth of strain-free, single-crystal islands of GaN on mechanically-exfoliated flakes of WS_2_ and MoS_2_, discussing their structural and optical properties. We also present a detailed comparison of temperature-dependent PL of GaN grown on WS_2_ and MoS_2_ and a preliminary demonstration of large-area growth of GaN on CVD MoS_2_. Our investigations establish TMDCs as interesting near-lattice-matched substrates for GaN. With appropriate choice of substrates and growth conditions, it opens up the prospect of combining the III-nitrides with the transition metal dichalcogenides to realize novel heterostructures such as stacked layered MoS_2_ and nitrides for solar energy conversion, as theoretically predicted^30^ recently. Further, growth on large-area single crystal TMDCs may provide a route to large-area single crystal GaN layers which could be released to serve as bulk substrates.

## Methods

### Preparation of substrates

All the TMDCs were synthesized by first reacting the constituent elements in stoichiometric ratio to form precursors followed by iodine vapour transport in order to get bulk crystals suitable for exfoliation into thin films (details in supplementary information section S2). In the case of MoS_2_, we used naturally available bulk MoS_2_ crystals for exfoliation. Raman spectroscopy details of the exfoliated and CVD grown MoS_2_, and exfoliated WS_2_ substrate materials are provided in supplementary information section S3.

### Growth of GaN layer

GaN layers were deposited on these TMDC substrates using low pressure MOVPE in a 3 × 2″ close-coupled showerhead system using standard trimethylgallium (TMGa) and NH_3_ precursors. H_2_ carrier gas was used for the GaN layer growth with samples being heated up and cooled down using N_2_ carrier gas. Based on a few flakes that we have studied (these are naturally a random selection) either via electron microscopy or SIMS, the growth rate of GaN layer on MoS_2_ is approximately 50–70 nm per minute.

### Characterization techniques

The films were structurally characterized using field-emission scanning electron microscopy (FE-SEM), X-ray diffraction (XRD), transmission electron microscopy (TEM) and electron back scattering diffraction (EBSD). The optical properties were measured by photoluminescence (PL) spectroscopy. Temperature-dependent PL measurements were done in the 10 K–295 K range using a set-up with a frequency-quadrupled 266 nm Nd:YAG laser for excitation, and a 0.55 m monochromator equipped with a cooled Si-CCD detector. Confocal Raman spectroscopy measurements were performed on the samples using 532 nm laser excitation. The Raman peak shift and the full width half maxima (FWHM) are determined by fitting a Lorentzian function to the observed data. For comparing the peak positions, the spectrum is aligned with reference to the Si substrate peak (520 cm^−1^).

## Additional Information

**How to cite this article**: Gupta, P. *et al.* Layered transition metal dichalcogenides: promising near-lattice-matched substrates for GaN growth. *Sci. Rep.* **6**, 23708; doi: 10.1038/srep23708 (2016).

## Supplementary Material

Supplementary Information

## Figures and Tables

**Figure 1:**
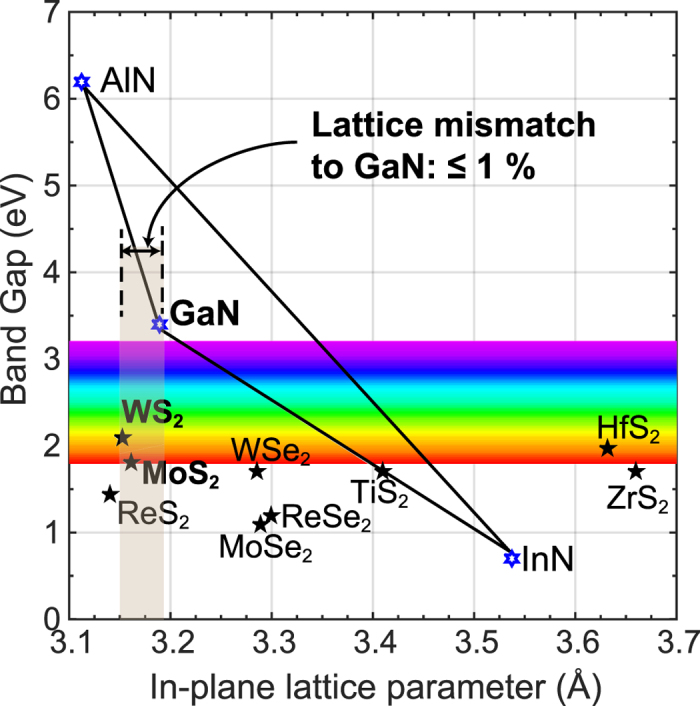
Band gap versus in-plane lattice parameter for different III-nitrides and TMDCs. The lattice mismatch of WS_2_ and MoS_2_ with respect to GaN are 1.0% and 0.8%, respectively. Supplementary information section S1 lists the sources from where the lattice parameters and bandgaps of different III-nitrides and TMDCs materials were obtained.

**Table 1:** The parameters E(0), a_*B*_, *θ*, Γ_i_ and *E*_i_ of GaN layers grown on WS_2_ and MoS_2_ (with 95% confidence bounds).

GaN/substrate	E(0) (eV)	a_*B*_ (meV)	*θ* (K)	Γ_i_ (meV)	*E*_i_ (meV)
GaN/WS_2_	3.468 ± 0.002	78 ± 18	365 ± 48	55 ± 16	20 ± 5
GaN/MoS_2_	3.469 ± 0.002	87 ± 24	385 ± 56	171 ± 87	46 ± 9
