# Supplementary material for: Layered transition metal dichalcogenides: promising near-lattice-matched substrates for GaN growth
Source: Sci Rep. 2016 Mar 30;6:23708. doi: 10.1038/srep23708 (PMC4812325; doi:10.1038/srep23708)
Supplement: Supplementary Information [file srep23708-s1.pdf]

Layered transition metal dichalcogenides: promising near lattice-matched substrates for GaN growth (Supplementary information)

Priti Gupta,[1,a] A. A. Rahman,[1] Shruti Subramanian,[1,b] Shalini Gupta,[1,2] Arumugam Thamizhavel,[1] Tatyana Orlova,[3] Sergei Rouvimov,[3] Suresh Vishwanath,[3,c] Vladimir Protasenko,[3] Masihhur R. Laskar,[4] Huili Grace Xing,[3,c] Debdeep Jena,[3,c] and Arnab Bhattacharya[1,d]

[1] *Department of Condensed Matter Physics and Materials Science, Tata Institute of Fundamental Research, Mumbai, India*

[2] *UM-DAE Center for Excellence in Basic Sciences, Mumbai, India*

[3] *Department of Electrical Engineering, University of Notre Dame, Notre Dame, USA*

[4] *Department of Chemical and Biological Engineering, University of Wisconsin-Madison, USA*

(Dated: 21 January 2016)

[a] Presently at Department of Materials Science and Metallurgy, University of Cambridge, Cambridge, UK

[b] Presently at Department of Materials Science and Engineering, Pennsylvania State University, Pennsylvania, USA

[c] Presently at Department of Electrical and Computer Engineering, Department of Materials Science and Engineering, Cornell University, New York, USA

[d] Email: arnab@tifr.res.in

S1. LATTICE PARAMETERS AND BANDGAP VALUES OF TMDCS AND III-NITRIDES

The bandgap versus lattice parameter diagram (Figure 1 of the main manuscript) was plotted based on the room temperature bandgap values of the monolayers of the TMDC materials as available in the literature

III-nitrides

- **AlN:** M. Feneberg, M. F. Romero, B. Neuschl, K. Thonke, M. Röppischer, C. Cobet, N. Esser, M. Bickermann and R. Goldhahn, *Applied Physics Letters* **102** (2013), 052112.
- **GaN:** B. Monemar, *Physical Review B* **10** (1974), 676.
- **InN:** J. Wu, W. Walukiewicz, K. Yu, J. Ager Iii, E. Haller, H. Lu, W. J. Schaff, Y. Saito and Y. Nanishi, *Applied Physics Letters* **80** (2002), 3967–3969.

TMDCs

- **WS_2:** A. Berkdemir, H. R. Gutiérrez, A. R. Botello-Méndez, N. Perea-López, A. L. Elías, C.-I. Chia, B. Wang, V. H. Crespi, F. López-Urías, J.-C. Charlier *et al.*, *Scientific Reports* **3** (2013).
- **MoS_2, $MoSe_2$:** S. Tongay, J. Zhou, C. Ataca, K. Lo, T. S. Matthews, J. Li, J. C. Grossman and J. Wu, *Nano letters* **12** (2012), 5576–5580.
- **WSe_2:** P. Tonndorf, R. Schmidt, P. Böttger, X. Zhang, J. Börner, A. Liebig, M. Albrecht, C. Kloc, O. Gordan, D. R. Zahn *et al.*, *Optics express* **21** (2013), 4908–4916.
- **$ReSe_2$:** C. Ho, P. Liao, Y. Huang, T. Yang and K. Tiong, *Journal of Applied Physics* **81** (1997), 6380–6383.
- **ReS_2:** S. Tongay, H. Sahin, C. Ko, A. Luce, W. Fan, K. Liu, J. Zhou, Y.-S. Huang, C.-H. Ho, J. Yan *et al.*, *Nature Communications* **5** (2014).
- **HfS_2:** D. L. Greenaway and R. Nitsche, *Journal of Physics and Chemistry of Solids* **26** (1965), 1445–1458.
- **TiS_2:** Y.-H. Liu, S. H. Porter and J. E. Goldberger, *Journal of the American Chemical Society* **134** (2012), 5044–5047.
- **ZrS_2:** M. Moustafa, T. Zandt, C. Janowitz and R. Manzke, *Physical Review B* **80** (2009), 035206.

The lattice parameters were obtained from their respective powder diffraction file in the database of Joint Committee on Powder Diffraction Standards (JCPDS), International Center for Diffraction Data, Newtown Square, PA (2013). The following is the list of JCPDS card numbers in the database:

III-nitrides

- **AlN:** 00-025-1133
- **GaN:** 00-050-0792
- **InN:** 00-050-1239

TMDCs

- **WS_2:** 04-003-4478
- **WSe_2:** 00-038-1388
- **MoS_2:** 00-037-1492
- **$MoSe_2$:** 04-004-8782
- **ReS_2:** 04-002-2231
- **$ReSe_2$:** 00-050-0537
- **HfS_2:** 00-028-0444
- **ZrS_2:** 00-011-0679
- **TiS_2:** 01-070-6204

S2. PREPARATION OF TRANSITION METAL DICHALCOGENIDES (TMDC) SUBSTRATES

Transition metal dichalcogenides (TMDC) of the type MX_2 where M is a transition metal and X is a chalcogen, are 2D layered materials, with van der Waals forces coupling the layers[1,2]. They can thus be easily exfoliated into thin monolayer to few-layer sheets, similar to graphene. We first prepared the TMDC crystals by the direct synthesis from their constituent elements. These crystals were exfoliated to get flakes that were transferred to a SiO_2/Si wafer as substrates for GaN growth. In the case of MoS_2, we used naturally available bulk MoS_2 crystals for exfoliation.

A. Crystal growth of TMDCs

A two-step process was followed for the crystal growth of the MX_2 (M= Mo, W, Re and X= S, Se). First the precursors for the crystal growth were prepared by the usual solid-state reaction of a stoichiometric mixture of the respective high purity constituent elements in an evacuated and sealed quartz ampoule ($\sim 10^{-5}$ torr). This quartz ampoule was placed inside a carbolite box furnace, which was slowly ramped to the reaction temperature at the rate of 15 - 20 ^{o}C/hr and kept at this temperature (1000 - 1200 ^{o}C) for 3 - 4 days , then cooled down to room temperature at 50 - 60 ^{o}C/hr. This resulted in flakes of the chalcogenide materials, typically 10 - 100 μm in size being synthesized. This product was then used in a second step to grow single crystals by the iodine vapor transport method. The reacted product ($\sim$ 1 g) along with I_2 (150 mg) were inserted in a 20 cm long, cleaned quartz tube, sealed and placed inside a 2-zone furnace. An appropriate temperature gradient ($\sim$ 100 - 120 ^{o}C) was maintained across the tube for a period of 4-8 days to enable the vapor transport and recrystallization of the material. Temperature parameters and duration during iodine vapor transport of different TMDCs are listed in Table 1.

TABLE I. Temperature parameters and duration during iodine vapor transport of different TMDCs

Initial material	Temperature Distribution		Growth Time (Days)
	Hot Zone (°C)	Cold Zone (°C)	
WS_2	950	850	7
MoS_2	1000	875	7
ReS_2	1020	975	8
WSe_2	800	700	4
$MoSe_2$	1080	1020	5
$ReSe_2$	1020	975	8

B. Exfoliation of TMDCs crystals

The TMDCs crystals obtained are then exfoliated using 3M scotch tape and stamped on 300 nm thick SiO_2-coated Si (~ 1 cm^2). This is then used as a substrate for the growth of GaN. Since our aim was to check the feasibility of the TMDC as a substrate for growth, we did not necessarily optimize the exfoliation to get monolayer sheets.

C. CVD growth of MoS_2

Large area MoS_2 films were synthesized by the vapor phase sulphurization of thin Mo films deposited on sapphire substrates[3]. Typically $\sim$ 10 - 15 nm thick Mo layers were allowed to

react with sulfur in an evacuated and sealed quartz tube at 900-1000 ^{o}C for 1 hour. Mo films deposited by sputtering and electron beam evaporation were both used with no significant difference in the quality of the resultant MoS_2.

S3. RAMAN SPECTROSCOPY MEASUREMENT OF MoS_2 AND WS_2 SUBSTRATES

Figure S1 shows the Raman spectra of the bare substrates, which clearly shows two prominent peaks E^1_{2g} and A_{1g}. The peak positions of these modes indicate the number of layers in the TMDCs[4,5]. The peak frequencies of E^1_{2g} and A_{1g} modes correspond to bulk MoS_2, both in case of exfoliated and CVD MoS_2. While in case of WS_2 which is prepared by exfoliating synthesized WS_2 bulk crystal, the number of layers varies from 3 to bulk all over the sample.

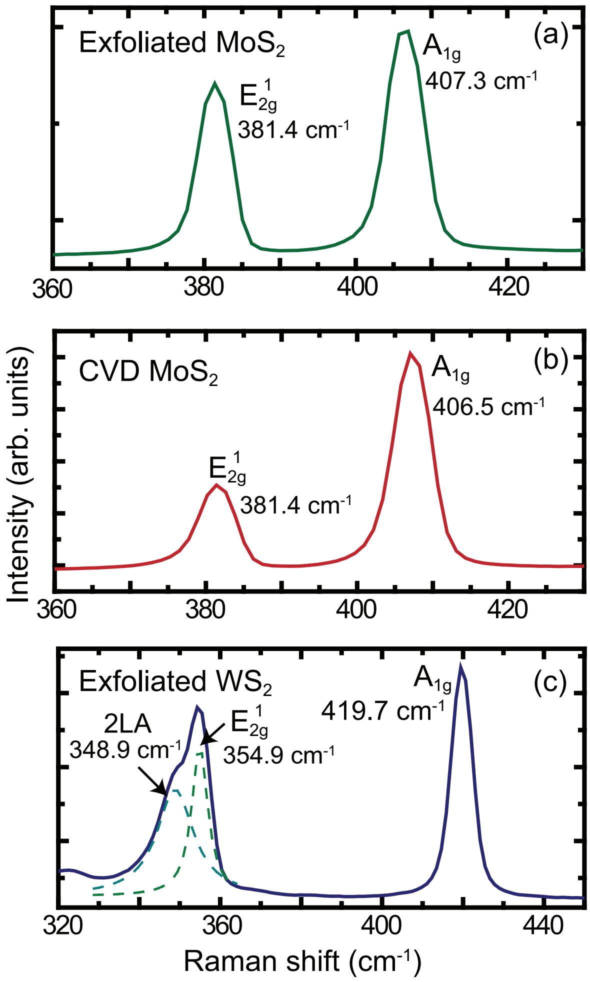

FIG. S1. **Raman spectra of bare substrates** (a) Exfoliated MoS_2 (b) CVD MoS_2 and (c) exfoliated WS_2 (dashed curves shows the Lorentzian two-peak fit for A_{2g} and 2LA modes).

Figure S2 shows the Raman spectra at different points on the hexagonal flake of GaN/WS_2 reported in Figure 2 of manuscript. It clearly shows the existence of WS_2 across the whole hexagonal flake. The black region between bright areas in the Raman integrated maps of 2LA and A1g modes (Figure 2 of manuscript) are just the regions with relatively low peak intensities. They appear even darker because the colour scale was chosen based on the (more intense) GaN E_2(high) peak.

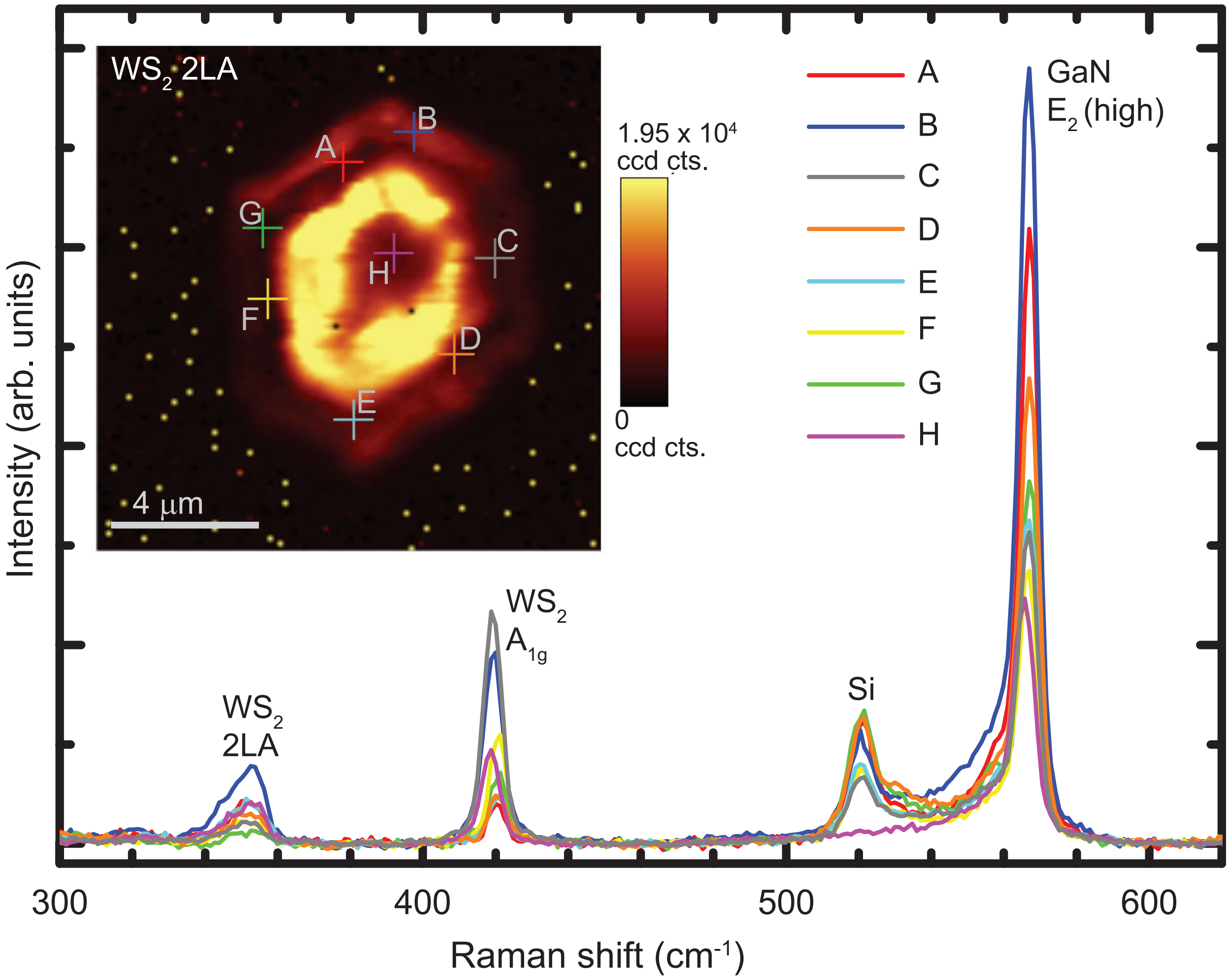

FIG. S2. **Raman spectra at different points on the hexagonal flake of GaN/WS_2 reported in Figure 2 of manuscript.** It clearly shows existence of WS_2 across the whole flake beneath the GaN.

S4. ANNEALING STUDIES ON MoS_2 AND WS_2

To check the survival of TMDCs in MOVPE growth conditions, we used the mechanically exfoliated TMDCs, which were transferred to 300 nm thick SiO_2-coated silicon wafers, and annealed at different temperatures (700 1040 ^{o}C) and in different gas ambients (H_2, NH_3, N_2). Figure S3 shows the representative SEM images of the WS_2 and MoS_2 before and after annealing. While we observed a change in morphology in TMDCs flakes after annealing in NH_3 and H_2 atmosphere, the flakes were intact after annealing at 900 ^{o}C in N_2 for 60 s. Also, no characteristic XRD peaks of the corresponding TMDC were observed in the samples, where morphology had changed after annealing. As an example, XRD profile of MoS2 before and after annealing is shown in Figure S4, where the MoS_2 peaks were not observed after annealing in ammonia at 1040 ^{o}C for 60 s, indicating that MoS2 had degraded while annealing. We believe that at high temperature, H_2 or atomic hydrogen from the decomposition of NH_3 reduces the sulphides to the metal.

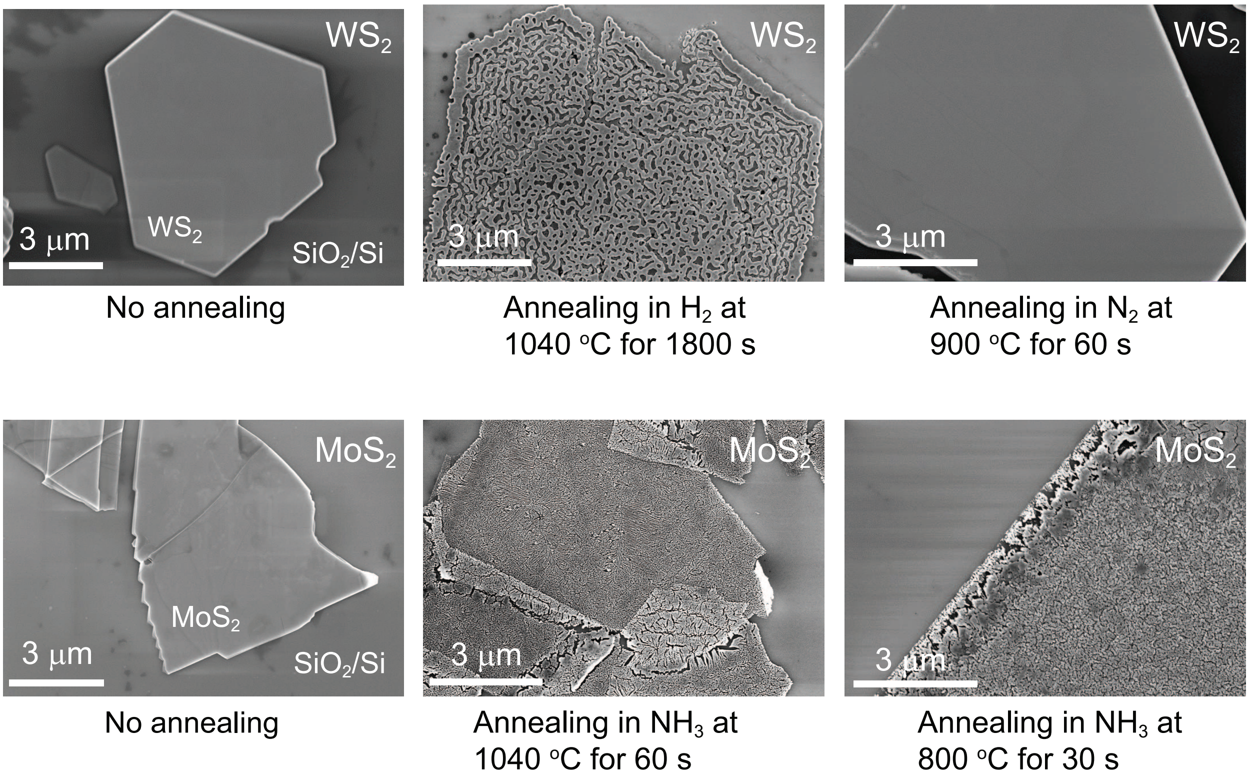

FIG. S3. **Representative SEM images showing the morphology of WS_2 and MoS_2 under different annealing conditions.** Exposure to high temperature, hydrogen and ammonia lead to change of morphology of WS_2 and MoS_2, making MOVPE growth of GaN on TMDCs difficult. However, there is no morphological change on annealing in N_2 at 900 ^{o}C for 60 s.

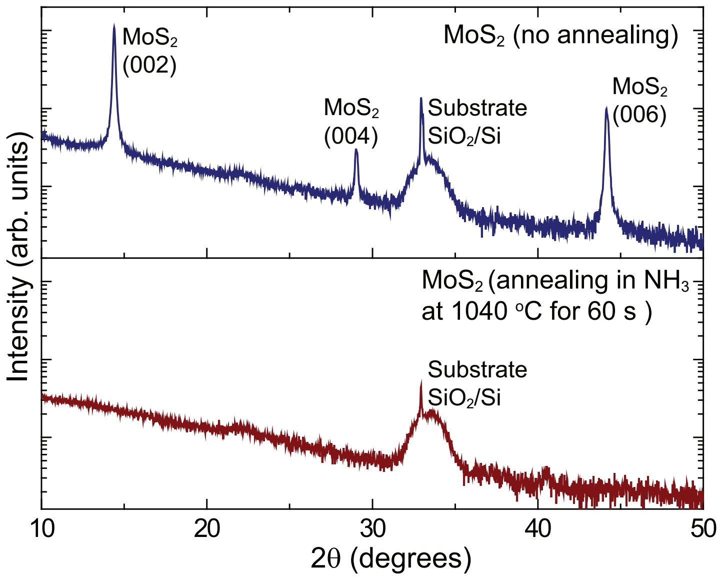

FIG. S4. **XRD profile of MoS_2 before and after exposure to MOVPE growth conditions.** X-ray diffraction shows no characteristic peaks of MoS_2 after annealing in ammonia at 1040 ^oC for 60 s, indicating degradation of MoS_2 while annealing.

Based on these observations, we optimized our growth conditions for GaN epitaxy on TMDCs. We used N_2 as the carrier gas instead of H_2 gas for ramping up the temperature and switched to H_2 simultaneously with introduction of precursors. An initial nucleation GaN layer was then grown then at 900 ^oC for 40 s to cover the TMDC layer and hence protect it from further exposure to NH_3 and H_2. Since the GaN layer quality is better at higher temperature, we then ramped up the temperature to 1040 ^oC for the further growth of GaN on TMDCs. With this procedure, we were able to protect WS_2 layer from degradation after MOVPE growth of GaN, however we need more optimization for the other TMDCs keeping in mind their different thermal stabilities.

S5. ELECTRON BACK SCATTER DIFFRACTION (EBSD) OF GaN/MoS_2

The EBSD map of GaN grown on exfoliated MoS_2 (Figure S5) clearly shows that grown GaN layer is single crystal similar to GaN grown on WS_2 and oriented in (0002) direction.

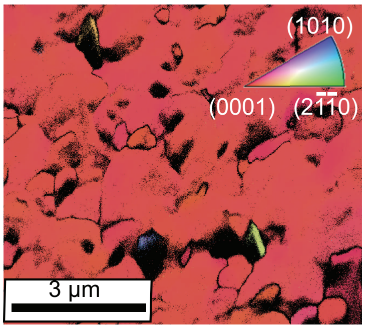

FIG. S5. **EBSD of GaN grown on exfoliated MoS_2** The map shows that GaN layer is single crystal and oriented in (0002) direction

S6. XRD PROFILE OF GaN GROWN ON CVD MoS_2

The XRD profile of GaN grown on CVD MoS_2 is similar to the XRD profile of GaN grown on exfoliated MoS_2.

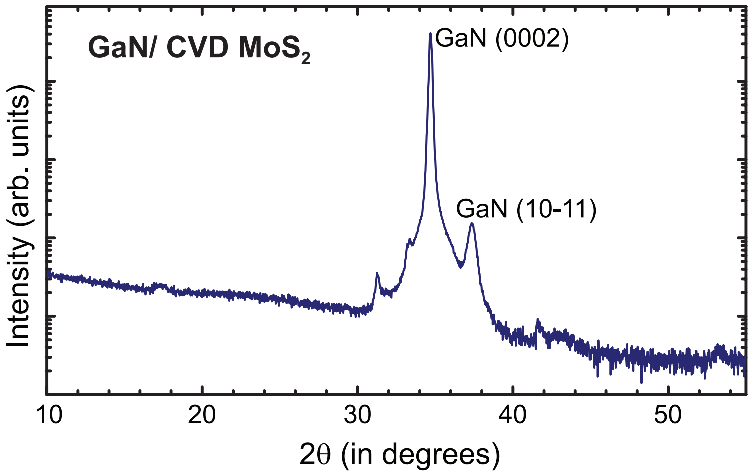

FIG. S6. **XRD profile of GaN layer grown on CVD MoS_2.** It is similar to the GaN layer grown on exfoliated MoS2 with no substrate peaks observed after the growth.

S7. SECONDARY ION MASS SPECTROMETRY (SIMS) OF GaN GROWN ON CVD MoS_2

As discussed in the main manuscript, MoS_2 was degrading after MOVPE growth of GaN. From an analysis of the behaviour of the photoluminescence of GaN on MoS_2 layers, we surmised that the degradation of MoS_2 left behind sulphur impurities in the layer. This was confirmed from the SIMS profile (done at Evans Analytical Group Inc.) which clearly shows a significant presence of sulphur in the GaN layer (Figure S7).

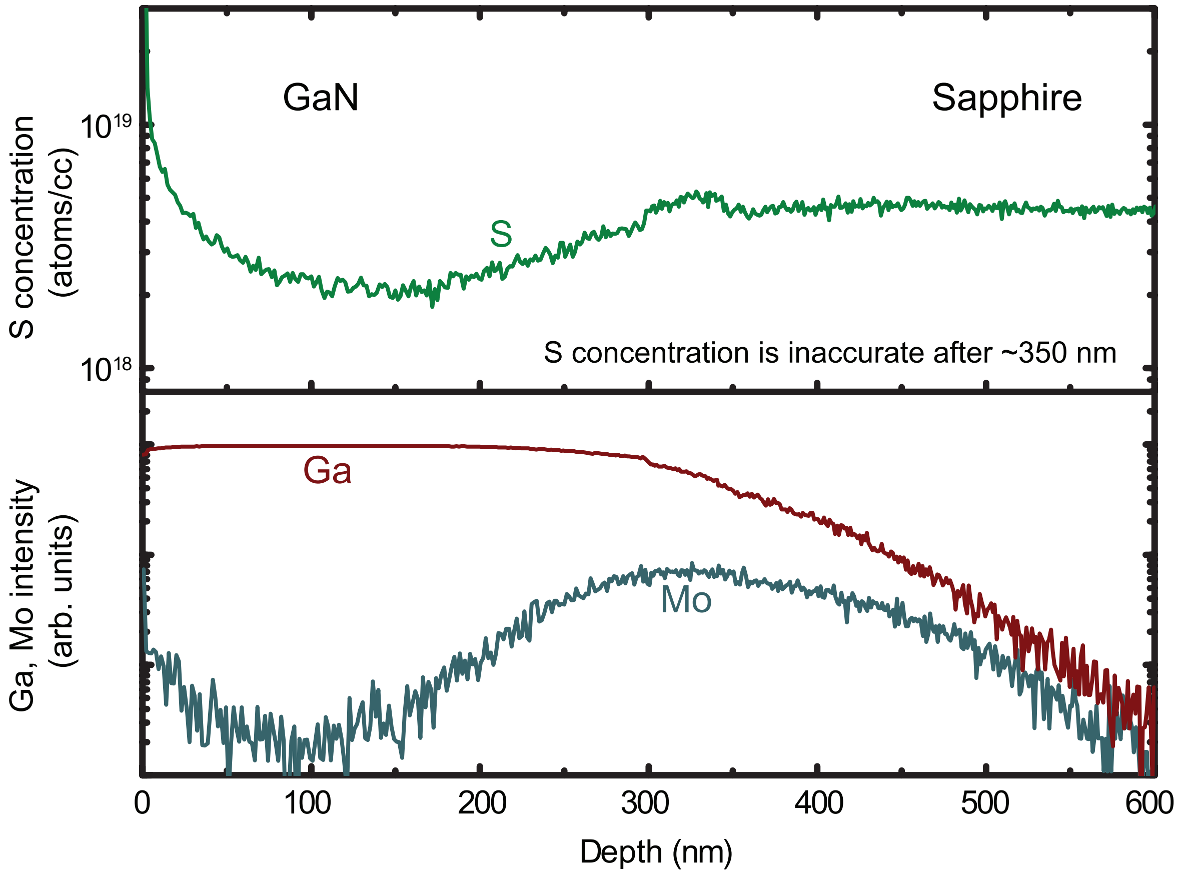

FIG. S7. **SIMS profile of GaN grown on CVD MoS_2/sapphire.** The profile shows significant presence of sulphur in the GaN layer.

S8. GaN GROWN ON OTHER TMDCs

Growth of GaN layers on other exfoliated TMDCs like WSe_2, $MoSe_2$, ReS_2 and $ReSe_2$ was also attempted and Figure S8 shows the corresponding SEM images. All these samples were grown following the same recipe used for the growth of GaN on WS_2 — a short initial layer at 900 ^oC, followed by 300 s growth at 1040 ^oC. From the micrographs, it is clear that the growth of GaN is possible on these substrates. However keeping in mind the different thermal stabilities of the various TMDCs, these conditions would need to be optimized independently for each material.

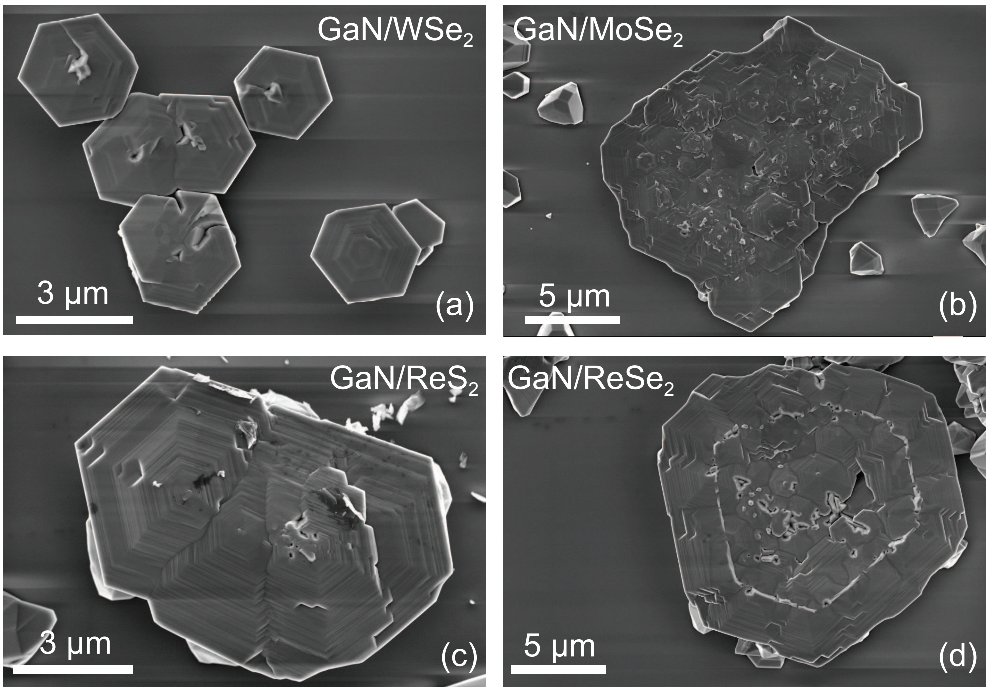

FIG. S8. **GaN grown on other TMDCs.** SEM images showing GaN grown on (a) WSe_2 (b) $MoSe_2$ (c) ReS_2 and (d) $ReSe_2$.

REFERENCES

[1]Neto, A. & Novoselov, K. New directions in science and technology: two-dimensional crystals. *Rep. Prog. Phys.* **74**, 82501–82509 (2011).

[2]Wang, Q. H., Kalantar-Zadeh, K., Kis, A., Coleman, J. N. & Strano, M. S. Electronics and optoelectronics of two-dimensional transition metal dichalcogenides. *Nature Nanotech.* **7**, 699–712 (2012).

[3]Laskar, M. R. *et al.* Large area single crystal (0001) oriented MoS_2. *Appl. Phys. Lett.* **102**, 252108 (2013).

[4]Berkdemir, A. *et al.* Identification of individual and few layers of WS_2 using Raman spectroscopy. *Sci. Rep.* **3** (2013).

[5]Li, H. *et al.* From bulk to monolayer MoS_2: evolution of Raman scattering. *Adv. Funct. Mater.* **22**, 1385–1390 (2012).
